# How often do German children and adolescents show signs of common mental health problems? Results from different methodological approaches – a cross-sectional study

**DOI:** 10.1186/1471-2458-14-229

**Published:** 2014-03-05

**Authors:** Kristin Sauer, Claus Barkmann, Fionna Klasen, Monika Bullinger, Gerd Glaeske, Ulrike Ravens-Sieberer

**Affiliations:** 1 Health Policy and Outcomes Research, Division Health Economics, Centre for Social Policy Research, University of Bremen, Mary-Somerville Straße 3, Bremen 28359, Germany; 2 Department of Child and Adolescent Psychiatry, Psychotherapy and Psychosomatics of the University Medical Center Hamburg-Eppendorf, Hamburg, Germany; 3 Institute and Polyclinic for Medical Psychology, University Clinic Hamburg-Eppendorf, Hamburg, Germany

## Abstract

**Background:**

Child and adolescent mental health problems are ubiquitous and burdensome. Their impact on functional disability, the high rates of accompanying medical illnesses and the potential to last until adulthood make them a major public health issue. While methodological factors cause variability of the results from epidemiological studies, there is a lack of prevalence rates of mental health problems in children and adolescents according to ICD-10 criteria from nationally representative samples. International findings suggest only a small proportion of children with function impairing mental health problems receive treatment, but information about the health care situation of children and adolescents is scarce. The aim of this epidemiological study was a) to classify symptoms of common mental health problems according to ICD-10 criteria in order to compare the statistical and clinical case definition strategies using a single set of data and b) to report ICD-10 codes from health insurance claims data.

**Methods:**

a) Based on a clinical expert rating, questionnaire items were mapped on ICD-10 criteria; data from the Mental Health Module (BELLA study) were analyzed for relevant ICD-10 and cut-off criteria; b) Claims data were analyzed for relevant ICD-10 codes.

**Results:**

According to parent report 7.5% (n = 208) met the ICD-10 criteria of a mild depressive episode and 11% (n = 305) showed symptoms of depression according to cut-off score; Anxiety is reported in 5.6% (n = 156) and 11.6% (n = 323), conduct disorder in 15.2% (n = 373) and 14.6% (n = 357). Self-reported symptoms in 11 to 17 year olds resulted in 15% (n = 279) reporting signs of a mild depression according to ICD-10 criteria (vs. 16.7% (n = 307) based on cut-off) and 10.9% (n = 201) reported symptoms of anxiety (vs. 15.4% (n = 283)). Results from routine data identify 0.9% (n = 1,196) with a depression diagnosis, 3.1% (n = 6,729) with anxiety and 1.4% (n = 3,100) with conduct disorder in outpatient health care.

**Conclusions:**

Statistical and clinical case definition strategies show moderate concordance in depression and conduct disorder in a German national sample. Comparatively, lower rates of children and adolescents with diagnosed mental health problems in the outpatient health care setting support the assumptions that a small number of children and adolescents in need of treatment receive it.

## Background

Child and adolescent mental health problems are ubiquitous and burdensome. Their contribution to functional disability and limited school activity [1], the high rates of accompanying medical illnesses [2] and the potential to last until adulthood [3] make them a major public health issue. The task of determining the magnitude of the problem is globally challenging. The lack of data gathering capacity for epidemiological studies is a major limitation [4]. Additionally, methodological factors lead to a variability in the estimates from epidemiological studies of mental health problems in children and adolescents.

These methodological factors are defined by problems of case definition, study design and epidemiological method used [5,6], when determining rates of mental health problems in children and adolescents. In addition to these factors that can cause a wide range of prevalence rates, other methodological issues have to be considered: (1) the inter-individual agreement is limited, pointing to the limited concordance of rater-specific results [7], (2) ICD-10 diagnoses of common mental health problems, such as depression, are developed and validated for adults, but applied to children and adolescents, and (3) the scoring criteria in questionnaire studies and the extent to which the criterion of functional impairment is taken into account [6-9]. As one of these listed factors, the case definition is a considerable methodological factor and operationalizing a mental health problem is generally based on mainly two different taxonomies: the statistical taxonomy that includes the dimensional classification of mental health problems derived from multivariate analyses on the one hand and the clinical taxonomy that is based on the categorical conception of mental health problems on the other hand. The statistical case definition is based on quantitatively scored items and syndromes that are reported by the informants and reflect the degree to which individuals manifest psychopathology. Subsequently, cut-off points distinguish between positive and negative screening results and display a threshold in order to avoid false positive results. However, based on lists of clinical symptoms and conditions, the clinical taxonomy using ICD-10 criteria was developed by the World Health Organization (WHO) to define mental health problems. The ICD-10 classification system is a conventionally used and internationally applied diagnostic guideline [10]. A review on the cross-cultural application of assessment and taxonomies of psychopathology showed small differences in problem rates and syndrome structure, comparisons indicate good overall concordance. Another review about psychopathology in childhood provided a comparison and emphasized the need for studying and building taxonomies [11,12]. The clinical and the statistical taxonomy do not converge to a degree that one approach can replace the other. Both approaches are needed and the combination of the two taxonomies is recommended when classifying mental health problems [13]. Moreover, both taxonomies have strengths and limitations.

Being labor intensive and time consuming, due to the complex appearance of mental health problems and the considerable likelihood of comorbidity, their definition requires a complicated diagnostic process, and only a small proportion of prevalence studies on population level are based on the clinical taxonomy using ICD-criteria. The majority of these studies are questionnaire studies as they are considered most efficient in large samples [6,8,14,15]. Earlier research has shown the limited generalizability of results from screening instruments due to the definition of cases according to cut-off points [15], and the need of standardized clinical interviews for the assessment of ICD or DSM diagnoses [6].

Several studies in different countries have assessed the prevalence of mental health problems in children and adolescents. A review of 52 studies that were carried out in over 20 countries mentioned the impact of the method used for case definition on prevalence rates. The mean estimate of overall prevalence was 15.8% and the median rates were 8% for preschoolers, 12% for preadolescents and 15% for adolescents. The majority of case definitions could be traced back to variations of the Rutter interview schedules or questionnaires. DSM-III and DSM-III-R criteria were also used frequently, but no studies reported ICD-10 criteria [5]. A more recent overview of the common disorders focused on studies that contain psychiatric diagnoses rather than distributional definitions of cases. Therein, anxiety was shown in 10.7% of adolescents between 12 and 19 years, depression in 6.1% and any of the behavioural disorders (conduct disorder, oppositional defiant disorder, or attention-deficit hyperactivity disorder) in between 3% and 4%. Most of these studies are based on the taxonomy of DSM-IIIR or DSM-IV, and one study was based on ICD-10 criteria [3].

In the reviews cited above, studies published in German were not taken into consideration, but have been systematically reviewed in an extensive meta-analysis in 2010. The results from prevalence studies of emotional and behavioural disorders in German children and adolescents are in line with international findings, with the majority of studies having been based on questionnaires. Accordingly, approximately every sixth child or adolescent shows symptoms of mental health problems [6].

The large population-based and nationwide sample in Germany (BELLA study) showed considerable parent-reported rates of mental health problems in German children and adolescents and the large impact on health-related quality of life [16]. Additionally, a poor parent-reported need for or provision of treatment was shown [9]. Based on results from questionnaires, 20.9% of the children and adolescents aged 7 to 17 years showed signs of overall mental health problems. Signs of specific mental health problems based on published cut-off points were present for conduct disorder in 7.6%, for depression in 5.4% and for anxiety in 10.0% of children aged 7 to 17 years [15]. The method of mapping questionnaire items to the diagnostic criteria was used for the study of prevalence rates of deficit-/hyperactivity disorder (ADHD) and hyperkinetic disorder (HD) according to DSM-IV criteria and ICD-10 criteria. The prevalence rates for the diagnosis of ADHD according to DSM-IV criteria were 5% and the frequency of HD according to ICD-10 criteria was 1%. These findings are in line with community studies in other countries [17]. With the majority of studies being based on questionnaires, there is a lack of prevalence rates for other commonly reported common mental health problems according to ICD-10 criteria.

Thus, methodological factors impact on the results, high rates of common mental health problems in children and adolescents are indicated, and there is a clear lack of prevalence rates according to ICD-10 criteria. Estimations of morbidity suggest that there is a large impact on the health related quality of life and poor awareness about need for treatment. International findings suggest only a small proportion of children with function impairing mental health problems are receiving treatment [8]. In Germany about 50% of persons with mental health problems show a need for treatment, and a considerably small number of children and adolescents with mental health problems are actually receiving treatment or are recognized as being in need of treatment [9,18]. But information about the health care situation of children and adolescents is scarce [19].

The presence of impairing psychopathology is related to service use suggesting that rates of service-contact can provide a proxy measure of the numbers of children who are most in need of intervention [20]. Thus, diagnoses from the outpatient health care setting are needed to reflect the actual health care utilization that might lead to a discussion about the extent of awareness of treatment needs of children and adolescents with mental health problems.

Against the background that methodologies should be carefully scrutinized in epidemiological studies and there is a lack of information on rates of mental health problems in children and adolescents according to ICD-10 criteria from nationally representative samples, the following research questions arise for this study:

1. How many children and adolescents meet the ICD-10 criteria according to the clinical definition of common mental health problems in a large population-based sample?

2. Do these results differ from analyses based on the statistical case definition of the same sample?

3. How many children and adolescents receive ICD-10 coded diagnoses in the outpatient health care setting?

This is the first approach on classifying common mental health problems, such as depression anxiety and conduct disorder, in German children and adolescents based on the clinical classification according to ICD-10 criteria from a nationwide representative sample in Germany. In addition, this study aims to add information about the utilization of health care.

## Methods

This cross-sectional study uses questionnaire data from the BELLA study and claims data of the statutory health insurance company Gmünder ErsatzKasse (GEK). In order to categorize children and adolescents based on disorder-related measures gained from questionnaire data of the BELLA study and their concordance with ICD-10 criteria, a clinical expert rating was conducted. Results from the statistical and clinical case definition strategies were compared on the basis of a single data set. Claims data of a statutory health insurance company (routine data) were analyzed for relevant ICD-10 codes according to sex and age. The reported diagnoses from routine data were clinically classified according to ICD-10-criteria and coded by health care professionals in the outpatient health care setting.

### Database

#### Primary data (Bella) - design, sample and instruments

For the Mental Health Module (BELLA study) of the National Health Interview and Examination Survey for Children and Adolescents of the Robert-Koch-Institute in Germany (KiGGS), 4,199 families were randomly selected from the KiGGS study´s nationwide representative sample of 17,641 families with children and adolescents aged 3 to 17 years. 68% of these families with children and adolescents aged 7 to 17 years agreed to participate and were interviewed. 2,863 children and adolescents were enrolled in the BELLA study and were asked to complete the telephone interview and paper questionnaires for parents of children aged 7 to 17 years and for adolescents aged 11 to 17 years. Data were weighted according to the age-, gender-, regional- and citizenship-structure of the German population. According to the weighted data, the population consisted of 1,467 males (51%) and 1,396 females (49%) from ages 7 to 17 years.

Of all males in the sample, 34% belonged to the age group of 7 to 10 and 66% to the group of 11 to 17 years. 33% of all females were between 7 and 10 years old and 67% belonged to the older age group.

The design and sample of the BELLA study are comprehensively described in Ravens-Sieberer et al. [21]. Data were collected between May 2003 and June 2006. Access to the BELLA-data is available on request. The ethics committee of the “Ärztekammer Hamburg” approved the use of this data base.

Anxiety, depression and externalizing problems were assessed using standardized screening instruments that account for diagnostic criteria according to the ICD-10 or DSM-IV. In order to determine the presence of anxiety disorders, the German version of the Screen for Child Anxiety Related Disorders – Questionnaire (SCARED) was completed by parents. The SCARED addresses panic/ somatic and generalized anxiety, separation anxiety, social and school phobia [22,23]. Information on depressive symptoms was obtained from the Center for Epidemiologic Studies Depression Scale (CES-D) [24,25]. Externalizing behavioral problems (delinquent and aggressive behavior) and suicidal tendencies were investigated with the German Version of the Child Behaviour Checklist (CBCL). Parents rated the behavior as “not true”, “somewhat or sometimes true” or “very true or often true”. The factorial validity and internal consistency of the German version of the CBCL has been proven [26-28]. Items of the Kidscreen_27 were applied for aspects of the quality of life (QoL) in children and adolescents [29] and items of the Conner´s Rating Scales-Revised were applied for aspects of hyperactivity or attention deficits [30].

#### Secondary data (claims data)

The basis for the secondary data analysis is pseudo-anonymous claims data of the year 2006 of a nation-wide German statutory health insurance company (Gmünder Ersatzkasse [GEK]^a^) with about 1.6 million insurants, which corresponds to approximately 2% of the German population. In Germany, about 85.9% and almost 70 million people were members of the statutory health insurance system in 2006 [31]. About 14% of the population were not insured with the statutory health insurance, but privately or with a composition of state-subsidy and private insurance.

The percentage of GEK insurants in the German population of the sixteen German states (Bundesländer) was between one percent (Sachsen-Anhalt) and three percent (Saarland) in 2006 [32]. Regarding it´s quantity, this sample exceeds all other German population based samples at this point, but it represents persons of only one health insurance fund in Germany. For this study, approximately 215,133 children and adolescents, 110,121 (51%) males and 105,012 (49%) females were analyzed. From all males and females 34% belonged to the age group of 7 to 10 and 66% to the group of 11 to 17.

Cases were selected when at least one of the relevant diagnoses were documented during one year (2006). Diagnoses were made by physicians and specialists in the outpatient health care setting according to the ICD-10 criteria. In Germany, physicians are legally obligated to keep the appropriate ICD-10 codes in the claims records. After a period of three month, the physician sends the claims data to the German association of statutory health insurance physicians (KBV) where they are checked for comprehensiveness and plausibility. According to the guidelines "Good Practice Secondary Data Analysis" (GPS) of the Working Group for the Survey and Utilization of Secondary Data (AGENS) and the Working Group for Epidemiological Methods, the analysis of routine data (secondary data) does not require an approval of an ethics committee, because data protection provisions on pseudo-anonymization of all personal data are fulfilled and there is no link to primary data.

### Rating

In order to validate the presentation of ICD-10 criteria by collating questionnaire items, specialized and clinically experienced (child and adolescent) psychiatrists rated the concordance of questionnaire items with diagnostic criteria of the ICD-10 classification. A total of four clinical experts participated in the rating. To assure standardization of the rating, raters followed a detailed rating-protocol. Subsequently, clinical experts rated the items regarding their accordance with the ICD-10 criterion. Each item was rated on a five-point Likert scale with 1 = “incongruity”, 2 = “little concordance”, 3 = „moderate concordance”, 4 = “good concordance” or 5 = “very good concordance”. Items were excluded for the presentation of ICD-10 criteria when the concordance was rated as “1” by at least two raters. In cases of missing items, experts were asked to add possible items from a list of all questionnaire items applied in the BELLA study.

Subsequently, children and adolescents were selected as cases, if they fulfilled the disorder related criteria of the ICD-10 classification scheme according to the following algorithms: For the definition of a single ICD-10 criterion, one of the associated items should be present. For the definition of depression according to ICD-10 criteria, at least two of the three symptoms specified under B must be present. An additional symptom or symptoms from the list “C” should be present, to give a total of at least four (mild depression), six (moderate depression) or eight (severe depression). A fifth character may be used to specify the presence or absence of the "somatic syndrome" (results not shown). For the definition of anxiety, recurrent anxieties or worries should be present according to Table 1. The classification of a conduct disorder followed the presence of three or more symptoms from the criterion list, of which at least three must be from items 9-24. The quoted items should be present with a rating of “predominantly true” or “especially true” (CES-D), ”Not true”, “somewhat true” in positive statements (e.g. “I/My child was happy”, CES-D) or “Somewhat true”, “predominantly true” or “especially true” (SCARED, CBCL). Tables 1, 2, 3 show disorder related ICD-10 criteria and associated questionnaire items.

**Table 1:** ICD-10 criteria for depression and associated questionnaire items

**ICD-10-criteria depressive episode F32**	**Item (abbreviated) “last week …“:**
**B.**	
(1) Depressed mood to a degree that is definitely abnormal for the individual, present for most of the day and almost every day, largely uninfluenced by circumstances, and sustained for at least 2 weeks.	(1) “My child could not shake of the blues even with help from family or friends”.
	“My child felt depressed“.
	“My child talked less than usual“.
(2) Loss of interest or pleasure in activities that are normally pleasurable;	(2) “My child was happy“.
	“My child talked less than usual“.
	“My child enjoyed life“.
(3) Decreased energy or increased fatiguability.	(3) “My child has been physically active (e.g. running)“ (negative response)
	“My child felt that everything he/she did was an effort“.
	“My child talked less than usual“.
	“My child could not get „going“.
**C**.	
(1) Loss of confidence and self-esteem;	(1) “My child felt it was just as good as other people.“ (negative response)
	“My child thought his/her life had been a failure.“
	“My child felt lonely.“
	“My child felt that people dislike him/her.“
(2) Unreasonable feelings of self-reproach or excessive and inappropriate guilt;	
(3) Recurrent thoughts of death or suicide, or any suicidal behavior;	(3) During the last 6 month my child:
	“My child hurt himself or attempted suicide“.
	“My child talked about suicide“
(4) Complaints or evidence of diminished ability to think or concentrate, such as indecisiveness or vacillation;	(4) “My child had trouble keeping his/her mind on what he/she was doing“.
	“During the last month, my child was inattentive and distractible.“
(5) Change in psychomotor activity, with agitation or retardation (either subjective or objective);	(5) *retardation:*
	“My child could not get going“.
	“My child talked less than usual“.
	“My child has been physically active (e.g. running) “ (negative response)
	*agitation:*
	“During the last month my child was twitchy“.
(6) Sleep disturbance of any type;	(6) “My child ´s sleep was restless“.
	“My child has nightmares about something bad happening to his/her parents“.
	“My child has nightmares about something bad happening to him/her“.
(7) Change in appetite (decrease or increase) with corresponding weight change).	(7) “My child did not feel like eating; appetite was poor“.

**Table 2:** ICD-10 criteria for anxiety and associated questionnaire items

**ICD-10 criteria generalized anxiety disorder in childhood F93.80**	**Item (abbreviated)**
(1) Excessive concerns about the quality of one's performance in areas such as schoolwork, sports, and other regular activities.	(1) “My child worries about things working out for him/her“.
	“My child had the idea that everything went wrong”.
	“My child felt it was just as good as other people“.
	“My child worries about being as good as other kids”.
	„My child worries about how well he/she does things“.
	“My child feels nervous when he/she is with other children or adults and he/she has to do something while they watch him/her (for example: read a loud, speak, play a game, play a sport)
	“My child worries about going to school”.
	“My child is scared to go to school”.
(2) Excessive concerns about physical health (despite an evident good health, or, if hurt or sick, concerns that go beyond a normal apprehension) or about being injured.	(2) “My child fells like he/she is going crazy when he/she gets frightened”.
	“My child is afraid of having anxiety (or panic) attacks”.
(3) Excessive concerns or anticipatory worries in relation to particular non-health themes (money or financial well-being, punctuality, appearance, catastrophes, disasters, etc.).	(3) “My child has nightmares about something bad happening to his/her parents“
	“My child worries about going to school.” “My child has nightmares about something bad happening to him/her“.
	“My child worries that something bad might happen to his/her parents”.
	„My child worries about what is going to happen in the future“.
	„My child worries about things working out for him/her“.
(4) Free floating anxiety unrelated to specific situations.	(4) “My child worries about going to school”.
	„My child worries about things working out for him/her“.
	„My child is a worrier“.
	“My child gets really frightened for no reason at all”.
	„People tell me that my child worries too much“.
	“My child is afraid of having anxiety (or panic) attacks”.
	“My child worries about what is going to happen in the future”.
	“My child is scared to go to school”.
	„ My child worries about things that have already happened“.
	“My child felt fearful.”
(5) A frequent need for reassurance that persists in spite of several appropriate attempts to reassure the child.	(5) „My child follows his/her mother/father wherever they go“.
	“My child feels nervous with people he/she does not know well”.
	„My child worries about things working out for him/her“.
	“My child feels nervous when he/she is going to parties, dances, or any place where there will be people that he/she does not know well”.
(6) Marked feelings of tension, inability to relax or to concentrate, nervousness, difficulty getting to sleep, autonomic symptoms (such as palpitations, sweating, dry mouth, etc.).	(6) “When my child gets frightened, it is hard for him/her to breathe”.
	“When my child gets frightened, he/she feels like passing out”.
	“My child is nervous“.
	“People tell me my child looks nervous“.
	“When my child gets frightened, his/her heart beats fast”.
	“When my child gets frightened, he/she gets shaky”.
	“When my child gets frightened, he/she gets sweats a lot”.
	“When my child gets frightened, he/she feels like he/she is choking”.
	“When my child gets frightened, he/she feels like throwing up”.
	“When my child gets frightened, he/she feels dizzy”.
	“My child had trouble keeping his/her mind on what he/she was doing“.
	“During the last month, my child was inattentive and distractible“.
(7) Recurrent somatic complaints (headaches, stomachaches, etc.) for which no physical basis can be demonstrated.	(7) “My child gets headaches when he/she is at school”.
	“My child gets stomachs at school”.

**Table 3:** ICD-10 criteria for conduct disorder and associated questionnaire items

**ICD-10-criteria conduct disorder F91**	**Item (abbreviated) “During the last 6 month my child…:”**

(1) Unusually frequent or severe temper tantrums for the child's developmental level.	(1) “…screamed a lot“.
	“…had temper tantrums or hot temper“.
	“…During the last month my child had temper“.
	tantrums or acted unpredictable“.
(2) Often argues with adults.	(2) “…argues a lot“.
(3) Often actively defies or refuses adults' requests or rules.	(3) “…is disobedient at home“.
	“…is disobedient at school“.
(4) Often, apparently deliberately, does things that annoy other people.	(4) “…doesn`t seem to feel guilty after misbehaving“.
(5) Often blames others for one's own mistakes or misbehavior.	(5) “…doesn´t feel guilty after misbehaving”.
(6) Often touchy or easily annoyed by others.	(6) “…got in many fights“.
	“… attacked people“.
	“… was stubborn, sullen, or irritable“.
	“… was irritable and impulsive“.
(7) Often angry or resentful.	(7) “…screamed a lot“.
	“… was stubborn, sullen, or irritable“.
(8) Often spiteful or vindictive.	(8) “…showed cruelty, bullying, or meanness to others“.
	“…destroyed things belonging to his/her family or others“.
	“…teases a lot“.
	“…showed vandalism“.
(9) Frequent and marked lying (except to avoid abusive treatment).	(9) “…lied or cheated“.
(10) Excessive fighting with other children, with frequent initiation of fights (not including fights with siblings).	(10) “…got in many fights“.
(11) Uses a weapon that can cause serious physical harm to others (e.g. a bat, brick, broken bottle, knife, gun).	
(12) Often stays out after dark without permission (beginning before 13 years of age).	
(13) Physical cruelty to other people (e.g. ties up, cuts or burns a victim).	(13) “…showed cruelty, bullying, or meanness to others.“
	“…my child attacked people“
	“…threatened people.“
(14) Physical cruelty to animals.	
(15) Deliberate destruction of others' property (other than by fire-setting).	(15) “…destroyed things belonging to his/her family or others.“
	“…showed vandalism.“
(16) Deliberate fire-setting with a risk or intention of causing serious damage.	(16) “…set fires.“
(17) At least two episodes of stealing of objects of value (e.g. money) from home (excluding taking of food).	(17) “…stole at home.“
(18) At least two episodes of stealing outside the home without confrontation with the victim (e.g. shoplifting, burglary or forgery).	(18) “…stole outside the home.“
(19) Frequent truancy from school beginning before 13 years of age.	(19) “…truancy, skips school“
(20) Running away from home (unless this was to avoid physical or sexual abuse).	(20) “…ran away from home.“
(21) Any episode of crime involving confrontation with a victim (including purse snatching, extortion, mugging).	(21) “…showed cruelty, bullying, or meanness to others.“
	“…child attacked people“
	“…stole outside the home.“
	“…threatened people.“
(22) Forcing another person into sexual activity against their wishes.	(22) “…showed cruelty, bullying, or meanness to others“.
	“…attacked people“.
	“…threatened people“.
(23) Frequent bullying of others (i.e. deliberate infliction of pain or hurt including persistent intimidation, tormenting, or molestation).	(23) “…showed cruelty, bullying, or meanness to others“.
	“…attacked people“.
	“…threatened people“.
(24) Breaks into someone else's house, building or car	(24) “…stole outside the home“.
Does not meet the criteria for dissocial personality disorder (F60.2), schizophrenia (F20.-), mania (F30.-), depression (F32.-), pervasive developmental disorder (F84.-), or hyperkinetic disorder (F90.-). (If criteria for emotional disorder (F93) are met, diagnose "mixed" disorder of conduct and emotions F92).	“My child does not have any mental health problem”.

### Statistical analysis

Statistical analyses of the primary data were based on weighted sample data according to the age-, gender-, regional- and citizenship-structure of the general German population.

Interviews including CES-D and SCARED questions about symptoms of depression and anxiety were conducted with 2,786 parents and 1,852 adolescents. Interviews which included CBCL questions about symptoms of externalizing problems were conducted with 2,452 parents. Cases in which persons did not complete the interview were therefore excluded from all analyses.

Problems of anxiety, depression and conduct disorder were described according to the ICD-10 classification scheme [10]. Therefore, symptoms and criteria relating to time and intensity were listed for generalized anxiety in childhood (F93.8), mild, moderate and severe depressive episode (F32) and conduct disorder (F91). Each symptom was presented by associated items from the questionnaires mentioned above. Subsequently, rates of mental health problems according to ICD-10 criteria are described as the percentages of agreement with the clinical symptoms and conditions defined by the associated criteria.

The established cut-offs were used for rates of mental health problems: Anxiety was measured with the German version of the SCARED-5 according to the specifications of Birmaher et al. (1999) with a cut-off of 3 [22]. The presence of depression was calculated using the cut-off point of 15 according to the American version of the Centre for Epidemiological Studies Depression Scale (CES-D) [25]. Symptoms of conduct disorder were classified according to values suggested in the Manual of the Child Behavior Checklist (CBCL) [26].

The intraclass-correlation-coefficient (ICC) was computed according to the Shrout and Fleiss notation from the rating of four study raters, with all subjects being rated by each rater, and reliability for the mean of k ratings (ICC(3,k)) [33].

Sex-specific differences were confirmed by p-values from computing chi-squared tests stratified by age. The Kappa statistic was calculated to show interrater-agreement between rates based on ICD-10 criteria and cut-offs and between parent- and self-reported rates [34].

Statistical analyses of the secondary data were based on the datasets of outpatient diagnoses and stem-information from the year 2006. These datasets were merged and analyzed on an individual basis. Children and adolescents were considered as cases if there was at least one of the following ICD-10 Codes documented during one year. For depression the ICD-10-codes “F32/33” were selected, for diagnoses of anxiety “F40/F41/F93.0, F93.1, F93.2, F93.8, F93.9” and for conduct disorder “F91”.

Analyses were conducted using the statistic package SAS (version 9.2).

## Results

The intraclass-correlation-analysis demonstrated moderate correlations of 0.6 for the mean of k ratings for the concordance of disorder related questionnaire items with diagnostic criteria of the ICD-10 classification. The ICD-10 criteria and associated questionnaire items that were selected are shown in Tables 1, 2, 3.

Based on parent report in all age groups 7.5% (n = 208) of children and adolescents fulfilled the criteria of the ICD-10 classification of a mild depressive episode and 11% (n = 305) showed symptoms of depression according to the cut-off score. 5.6% (n = 156) fulfilled the criteria of generalized anxiety and 11.6% (n = 323) scored above the cut-off. 15.2% (n = 373) fulfilled the criteria of a conduct disorder, whereas 14.6% (n = 357) showed signs of a conduct disorder based on cut-off. Based on self report, the age group of 11 to 17 showed signs of a mild depression according to ICD-10 criteria in 15% (n = 279) and according to cut-off in 16.7% (n = 307). 10.9% (n = 201) of the older age group fulfilled the criteria of a generalized anxiety and 15.4% (n = 283) scored above the cut-off according to self-report.

Results from the classification of depressive symptoms according to the ICD-10 criteria indicate an increase with age and a higher, but not statistically significant, proportion in females and a higher proportion based on self-report. Results based on the questionnaire cut-off show higher proportions in females and based on self-report as well. However, in the younger age group, the frequency of males showing signs of depression is higher than rates in females.

Results of generalized anxiety according to ICD-10 criteria show an increase in age and a higher proportion of females and based on self-report. Sex-specific differences are significant in the results based on self-report. Frequency based cut-offs are significantly higher in younger females and in rates based on self-report.

Rates of conduct disorder according to ICD-10 criteria are significantly higher in males (Table 4).

**Table 4:** Symptoms of common mental health problems according to ICD-10 criteria and the frequency of high symptom scores based on cut-off, by sex and age

	**ICD-10 criteria [95% CI]**		**Questionnaire cut-off [95% CI]**	
	***Male***	***Female***	***Male***	***Female***
***7-10 parent-report***				
Depressive episode	4.3 [2.4-6.2]	5.2 [2.7-7.8]	11.3 [8.3-14.3]	9.9 [6.7-13.2]
Generalized anxiety	3.6 [1.9-5.3]	4.1 [2.3-5.9]	12.5 [9.4-15.5]*	17.1 [13.2-20.9]
Conduct disorder	21.9 [17.8-26.1]*	12.2 [8.4-15.9]	16.0 [12.4-19.6]	14.9 [11.1-18.7]
***11-17 parent-report***				
Depressive episode	8.2 [6.1-10.3]	9.7 [7.2-12.1]	10.7 [8.2-13.3]	11.5 [8.9-14.2]
Generalized anxiety	7.6 [5.4-9.8]	5.4 [3.6-7.1]	8.7 [6.4-10.9]	11.3 [8.7-13.9]
Conduct disorder	17.3 [14.0-20.7]*	10.7 [7.9-13.5]	15.0 [11.9-18.2]	13.1 [10.2-16.0]
***11-17 self-report***				
Depressive episode	15.3 [12.4-18.2]	15.0 [12.1-17.8]	12.3 [9.6-15.0]*	21.2 [18.0-24.4]
Generalized anxiety	7.2 [5.1-9.4]*	14.7 [11.9-17.4]	11.8 [9.2-14.5]*	18.9 [16.0-22.0]

For depression ICD-10 criteria according to mild depressive episode are shown. *P < 0.05.

Rates of children and adolescents with symptoms of mild and moderate depression according to ICD-10 criteria increased with age, were over all higher in females and in rates based on self-report. Significant differences in sex-specific results are reported for rates of moderate depression based on self-report. Severe depression shows the lowest frequencies and higher rates in males based on parent-report, but no significant differences in sex-specific results (Table 5).

**Table 5:** Symptoms of mild, moderate and severe depression according to ICD-10 criteria, by sex and age

**ICD-10 criteria [95% CI]**		
	***Male***	***Female***
***7-10 parent-report***		
Mild	4.3 [2.4-6.2]	5.2 [2.7-7.8]
Moderate	3.1 [1.4-4.7]	2.8 [1.2-4.4]
Severe	0.9 [0.1-1.7]	0.8 [0.0-1.6]
***11-17 parent-report***		
Mild	8.2 [6.1-10.3]	9.7 [7.2-12.1]
Moderate	5.4 [3.7-7.1]	4.8 [3.1-6.4]
Severe	2.1 [0.9-3.3]	1.6 [0.6-2.6]
***11-17 self-report***		
Mild	15.3 [12.4-18.2]	15.0 [12.1-17.8]
Moderate	7.1 [5.2-9.1]*	10.1 [7.6-12.6]
Severe	0.8 [0.2-1.4]	1.8 [0.6-2.9]

*P < 0.05.

The results from analyzing interrater-agreement between the two case definition strategies in children and adolescents from the BELLA-sample showed different Kappa coefficients (Table 6). Moderate interrater-agreement was calculated for depression and conduct disorder. Fair interrater-agreement between the two case definition strategies is reported for anxiety in the older age groups and low agreement in the younger age group according to parent-report.

**Table 6:** Symptoms of common mental health problems according to ICD-10 criteria and high symptom scores based on cut-off and interrater-agreement

	**ICD-10 criteria [95% CI]**	**Questionnaire cut-off [95% CI]**	**Kappa**
***7-10 parent report***			
Depressive episode	4.8 [3.2-6.3]	10.6 [8.4-12.8]	0.5
Generalized anxiety	3.8 [2.6-5.1]	14.7 [12.3-17.2]	0.1
Conduct disorder	17.2 [14.3-20.0]	15.4 [12.8-18.1]	0.5
***11-17 parent-report***			
Depressive episode	8.9 [7.3-10.5]	11.1 [9.3-12.9]	0.6
Generalized anxiety	6.5 [5.1-7.9]	10.0 [8.2-11.7]	0.3
Conduct disorder	14.1 [11.9-16.3]	14.1 [11.9-16.3]	0.4
***11-17 self-report***			
Depressive episode	15.1 [13.1-17.2]	16.7 [14.6-18.8]	0.5
Generalized anxiety	10.9 [9.2-12.7]	15.4 [13.4-17.4]	0.3

For depression ICD-10 criteria according to mild depressive episode are shown.

Besides agreement between the two case definition strategies, agreement between parent- and self-report was analyzed for depression and anxiety according to ICD-10 criteria. Kappa coefficients below 0.2 showed only low agreements between parent and self-report.

Results from routine data indicate that 0.9% (n = 1,196) of children and adolescents with a depression diagnosis, 3.1% (n = 6,729) with a diagnosis of anxiety and 1.4% (n = 3,100) with conduct disorder based on ICD-10 codes from the outpatient health care setting. There is an increase in the percentage of children and adolescents with ICD-10 codes of depression with age, and the highest amount of 1.5% in females from the older age group. Anxiety slightly decreases with age and shows the highest proportion in younger males, whereas conduct disorder is more frequent in males. The sex-specific differences are significant, except for proportions of anxiety including all ages and depression in the younger age group (Table 7).

**Table 7:** Routine data: characteristics of children and adolescents with special mental health problems according to ICD-10 in 2006, by sex and age

**Routine data**	**Male**	**Female **
Diagnosis**		
Depressive episode (F32/ F33)	0.7% [0.7-0.8]*	1.1% [1.0-1.2]
Anxiety (F40/F41/F93.0/F93.1/F93.2/F93.8/F93.9)	3.1% [3.0-3.2]	3.1% [3.0-3.2]
Conduct disorder (F91)	2.0% [1.9-2.1]*	0.9% [0.8-0.9]
***7-10 years***		
Depressive episode (F32/ F33)	0.4% [0.3-0.5]	0.4% [0.3-0.5]
Anxiety (F40/F41/F93.0/F93.1/F93.2/F93.8/F93.9)	4.2% [4.0-4.4]*	3.5% [3.3-3.6]
Conduct disorder (F91)	2.8% [2.7-3.3]*	1.2% [1.1-1.3]
***11-17 years***		
Depressive episode (F32/ F33)	0.9% [0.8-0.9]*	1.5% [1.4-1.6]
Anxiety (F40/F41/F93.0/F93.1/F93.2/F93.8/F93.9)	2.5% [2.4-2.7]*	3.0% [2.9-3.1]
Conduct disorder (F91)	1.5% [1.4-1.6]*	0.7% [0.6-0.8]

*P < 0.05 **some children received more than one of these diagnoses.

## Discussion

Since determining rates of emotional and behavioural disorders in children and adolescents is associated with the problem of defining a disorder, estimates from international studies differ widely [3,8]. In general, the reported frequencies of children and adolescents showing signs of common mental health problems can be considered to be in line with previous research. Median estimates for anxiety were shown between 10,4% and 21%, highlighting that the identification does not necessarily correlate with the degree of impairment [4,8,36]. The median estimate for depression of 4,4% [15,36] can be reflected by the results from ICD-10 criteria regarding mild and moderate depressive episodes. Even though, results show the typical higher scores in boys on externalizing problems and in girls on internalizing problems, questionnaire methods applying symptom ratings are limited and might lead to overestimation [7]. The cut-off criteria of some screening instruments used for this study could be considered as too liberal, with the result of low specificity and overestimated rates of children with the common mental health problems. Especially the screening instruments used to identify specific mental health problems in population based studies could not yet be compared to a reliable gold-standard [15]. Our findings confirm earlier results on the similarities in self-reported results across different epidemiological approaches [4].

The comparison of results based on the two case definition strategies shows that the frequency of children and adolescents with signs of a conduct disorder according to ICD-10 criteria corresponds well to the reported rates based on a high symptom score. According to the Kappa statistic there is moderate concordance between frequencies according ICD-10 criteria and cut-offs in depression and conduct disorder. Respectively low rates according to ICD-10 criteria are shown in depression and anxiety based on parent report, which can be due to several reasons. Lower rates according to ICD-10 criteria were shown when results from ICD-10 criteria were compared to DSM-IV [17,37]. But obviously, only rates based on parent-report show differences between the two case definition strategies in depression and anxiety with lower rates according to ICD-10 criteria, whereas frequencies from self-report show stronger parallels. This might be due to the limited concordance of rater-specific results and the restricted information of parents especially about internalizing disorders, which are considered to be less obvious for parents than externalizing problems. Kappa coefficients suggest low agreement between parent- and self-report for depression and generalized anxiety according to ICD-10 criteria. This is in line with a study on depression according to cut-off criteria, which showed limited inter-individual agreement of parent- and self-report on depressive symptoms [7].

Estimates of overall mental health problems in children and adolescents from other German and international studies show an increase in the older age groups [35,38], which can be reflected in our results based on the parent-reported ICD-10 criteria. Though, unlike the typically higher prevalence rates of overall mental health problems in older children and adolescents, our results based on the parent reported cut-offs suggest a small decrease of the reported common mental health problems in the older age group. While self report shows higher rates in older children and adolescents, these findings from parent-report were shown earlier from the same sample [9], possibly due to parents reports being less sensitive regarding the mental health problems of older children and adolescents.

The parent reported rates of generalized anxiety according to ICD-10 criteria show higher rates in males in the older age group, whereas rates based on the published cut-off show higher rates in females in all age groups. In addition to the low interrater-agreement between parent- and self-report, it might be possible that signs of anxiety in boys are surprising and therefore more noticeable for parents according to the gender-specific prejudice of girls being more anxious. Also, results from routine data showed higher rates of anxiety in males, but in the younger age group.

Results from routine data showed relatively small percentages of children and adolescents with ICD-10 codes of depression, anxiety and conduct disorder. This might be due to poor awareness of children and adolescents in need for support and health care. Also, the low rates of ICD-10 codes for depression in children and adolescents might be due to the fact that the diagnosis of depression according to the ICD-10 criteria is neither developed nor validated for children and adolescents. The higher rates of females with signs of a depression and the increase with age can be seen in results from routine data, whereas rates of conduct disorder are higher in males and decrease with age.

However, in contrast to relatively high prevalence rates from primary data, the proportion of children having service contact appears small and the results suggest a considerable number of children and adolescents needing treatment that does not have contact to health care services [6]. Lack of information, uncertainties concerning the diagnosis, stigmatization, social factors and access to services or to primary prevention reflect factors that might affect service use by children and adolescents with mental health problems [39] and thereby limit the information gained from routine data. Only a few factors that might affect service use were investigated in recent research: In a systematic review of the prevalence and detection of depressive disorders in German general practice, low detection rates of depression-related diagnoses were found in general practice [40]. These results refer to adults. In order to meet treatment needs, it is highly desirable that children and adolescents with symptoms should be diagnosed adequately. In Germany, general health care for children is usually provided by paediatricians. Recent analyses on the outpatient treatment of depression in adolescents aged 12 to 18 years showed large numbers contacting general practitioners and pediatricians, but only one third with depression being seen by a child and adolescent psychiatrist [41].

This is the first representative German study on the classification of symptoms of common mental health problems in children and adolescents according to ICD-10 criteria. However, some limitations must be mentioned. Even though the survey items correspond well to the ICD-10-criteria, the reported rates of mental health problems are not equitable to clinical diagnoses. The presentation of some ICD-10 criteria by adequate questionnaire items, especially relating to time and particular criteria of conduct disorder, remains improvable. Some criteria shown in Tables 1, 2, 3 were imperfectly presented by questionnaire items since they were used to assess more than one criterion.

Other common diagnoses, such as bipolar disorder, schizophrenia or autism have not been evaluated and should be studied in future research. As shown for ADHD according to ICD-10 [17], comorbidity should be studied in depression, anxiety and conduct disorder in future research. The impact of rater-specific perspectives on rates of mental health problems was shown and should receive attention in upcoming studies. Parent-reported information might be less sensitive concerning internalizing mental health problems of adolescents, such as depression or anxiety, which might lead to an underestimation of the number of children and adolescents affected. In contrast, parent-reported information is highly sensitive concerning externalizing behavior, such as conduct disorder.

Information about diagnoses that is gained from routine data originates from the ICD-10 codification process in the outpatient health care setting. Not including the documentation of the diagnostic criteria, information on the precision of a physician´s diagnosis is lacking in claims data. Even though, the diagnostic process is based on international guidelines on the ICD-10 classification of mental health problems, uncertainties of the codification should be considered. In this study, ICD-10 codes from routine data provide a proxy measure for the health care utilization of children and adolescents with common mental health problems. While routine data do not contain the risks of recall-bias or Hawthorne effect, interpretations should consider a possible selection bias since the information gained should only be referred to those contacting the health care system. Besides, there are possible differences in the characteristics of insurants or health care utilization between members of different health insurance funds in Germany. Recent analyses have shown differences in the proportion of children and adolescents with migration background, somatic diseases and psychopathological problems between the different health care insurance funds [42].

A major strength of the present study is that it adds information on common mental health problems in German children and adolescents according to ICD-10 criteria, which are immensely labor- and time-consuming to investigate in large samples. This study examines some methodological challenges determining rates of common mental health problems in children and adolescents.

Data on children and adolescents from the outpatient health care setting are scarcely available to date. The claims data used for this study allow a precise calculation of utilization patterns from a nation-wide sample of the insured population. So far, health insurance data seem to be a promising source to detect particular population-based information. Prior studies showed that findings from GEK-data and from cross-sectional studies such as surveys and screenings show strong parallels in the prevalence of adult depression [43]. The quantity of the routine data analyzed in this study is a major strength. Not being vulnerable to recall-bias, routine data generally cover a large variety of persons that are often excepted from trials and epidemiological studies, e.g. by including all ages and institutionalized persons. Data, especially of this quantity, that reflect the actual health care situation of children and adolescents with mental health problems in the statutory health insurance system, are limited in Germany.

## Conclusions

The attempt at determining rates according to ICD-10 criteria using questionnaire items and comparing them to rates based on questionnaire-cutoffs showed that results strongly depend on the method used for defining cases. The low inter-individual agreement in ICD-10 criteria between parent- and self-report shows the need for multiple informants, whereas self-reported results confirm consistency across different epidemiological approaches.

On the one hand, the presentation of ICD-10 criteria via questionnaire items remains improvable, since in some cases more specific items need to be applied. Results based on questionnaires might to lead to an overestimation on the other hand. Thus, in order to validate the methodological approaches used in this study, it would be very useful to compare the two case definition strategies with a gold-standard like a valid diagnosis. Since considering methodological aspects of epidemiology of mental health problems is not adequate for an appreciation of understanding the burden of child and adolescent mental health problems, measuring functional impairment can provide important information on the (individual) burden. However, the results presented here provide insight to methodological aspects of the epidemiological study of mental health problems from population based samples that have not been scrutinized to date and present frequencies of children and adolescents showing clinical relevant symptoms that as a minimum call for further diagnostics.

Respectively low rates of children and adolescents with mental health problems in the outpatient health care setting support earlier assumptions of the small number of children and adolescents in need receiving treatment. Against the background that high proportions of children and adolescents with mental health problems are in need of treatment, and the stability of symptoms is known in particular disorders, there is a clear lack of research investigating factors that might affect health care service use in children and adolescents, such as uncertainties in the diagnosis and stigmatization. In order to meet treatment needs in different age- and sex-groups, it is highly desirable that children and adolescents with symptoms receive adequate diagnoses.

## Endnote

^a^Which is the BARMER GEK since 2010.

## Competing interests

The authors declare that they have no conflict of interest.

## Authors’ contributions

The work presented here was carried out in collaboration between all authors. URS conceived of the study, URS, KS and CB designed methods; KS analyzed the data, interpreted the results and wrote the paper. FK, CB, MB and GG participated in the design of the study and drafted the manuscript. All authors read and approved the final manuscript.

## Pre-publication history

The pre-publication history for this paper can be accessed here:

http://www.biomedcentral.com/1471-2458/14/229/prepub
